# Supplementary material for: Integrative spatial omics reveals distinct tumor-promoting multicellular niches and immunosuppressive mechanisms in Black American and White American patients with TNBC
Source: Nat Commun. 2025 Jul 17;16:6584. doi: 10.1038/s41467-025-61034-3 (PMC12271405; doi:10.1038/s41467-025-61034-3)
Supplement: Supplementary file 3 — Reporting Summary [file 41467_2025_61034_MOESM3_ESM.pdf]

nature portfolio

Corresponding author(s): Qian Zhu, Arun Sreekumar

Last updated by author(s): May 8, 2025

Reporting Summary

Nature Portfolio wishes to improve the reproducibility of the work that we publish. This form provides structure for consistency and transparency in reporting. For further information on Nature Portfolio policies, see our Editorial Policies and the Editorial Policy Checklist.

Statistics

For all statistical analyses, confirm that the following items are present in the figure legend, table legend, main text, or Methods section.

n/a	Confirmed	
☐	☒	The exact sample size (*n*) for each experimental group/condition, given as a discrete number and unit of measurement
☐	☒	A statement on whether measurements were taken from distinct samples or whether the same sample was measured repeatedly
☐	☒	The statistical test(s) used AND whether they are one- or two-sided<br>*Only common tests should be described solely by name; describe more complex techniques in the Methods section.*
☐	☒	A description of all covariates tested
☐	☒	A description of any assumptions or corrections, such as tests of normality and adjustment for multiple comparisons
☐	☒	A full description of the statistical parameters including central tendency (e.g. means) or other basic estimates (e.g. regression coefficient) AND variation (e.g. standard deviation) or associated estimates of uncertainty (e.g. confidence intervals)
☐	☒	For null hypothesis testing, the test statistic (e.g. *F*, *t*, *r*) with confidence intervals, effect sizes, degrees of freedom and *P* value noted<br>*Give P values as exact values whenever suitable.*
☒	☐	For Bayesian analysis, information on the choice of priors and Markov chain Monte Carlo settings
☐	☒	For hierarchical and complex designs, identification of the appropriate level for tests and full reporting of outcomes
☐	☒	Estimates of effect sizes (e.g. Cohen's *d*, Pearson's *r*), indicating how they were calculated

Our web collection on statistics for biologists contains articles on many of the points above.

Software and code

Policy information about availability of computer code

Data collection	The IMC data was collected using CyTOF software (version 7.1)-Standard BioTools. Spatial transcriptomics data was collected using Space Ranger (version 2.1.0)-10x Genomics, NovaSeq X (sequensor, instrument)- Illumina and OlyVIA 4.2- Olympus.The cell segmented data of IMC images in BSW discovery dataset was generated using MCD Viewer (version 1.0.560.6), CellProfiler (version 4.2.1) and Histocat(version 1.73). The cell segmented data of IMC images in BSW validation and Roswell Park dataset was generated using Steinbock software (version 0.16.3)
Data analysis	The codes used to analyze the TNBC disparity data in this study were developed using R 4.3, Python 3.10,ImageJ 1.54 and Shell 4.2.46. The codes used to analyze the data in this study are deposited in GitHub under the URL link https://qianzhulab.github.io/suppl/TNBC.scripts/

For manuscripts utilizing custom algorithms or software that are central to the research but not yet described in published literature, software must be made available to editors and reviewers. We strongly encourage code deposition in a community repository (e.g. GitHub). See the Nature Portfolio guidelines for submitting code & software for further information.

Data

Policy information about availability of data

All manuscripts must include a data availability statement. This statement should provide the following information, where applicable:

- Accession codes, unique identifiers, or web links for publicly available datasets
- A description of any restrictions on data availability
- For clinical datasets or third party data, please ensure that the statement adheres to our policy

Data Availability: All data supporting the findings of this study are available within the paper and its Supplementary Information. The 10X VisiumSpatial

Trancriptomic data of TNBC patients including the minimum dataset from the Georgia cohort has been deposited in Zenodo under the following URL: https://doi.org/10.5281/zenodo.12797059. The Nanostring GeoMx data has been deposited into Zenodo under the following URL: https://doi.org/10.5281/zenodo.12752405. The Imaging Mass Cytometry (IMC) data has been deposited into Zenodo under the following URL: https://doi.org/10.5281/zenodo.15115492. Source data are provided with this paper.
Code Availability: The codes used to analyze the TNBC disparity data in this study were developed using R, Python and Shell. The codes used to analyze the data in this study are deposited in GitHub under the URL link https://qianzhulab.github.io/suppl/TNBC.scripts/. DOI has been created from Github: 10.5281/zenodo.15353111

Research involving human participants, their data, or biological material

Policy information about studies with human participants or human data. See also policy information about sex, gender (identity/presentation), and sexual orientation and race, ethnicity and racism.

Reporting on sex and gender	The study focuses on Triple Negative Breast Cancer (TNBC) in females (people assigned female sex at birth). The data was available as a part of the metadata file associated with the samples.
Reporting on race, ethnicity, or other socially relevant groupings	For this study, race groups were self-reported Black American (BA) or White American (WA) TNBC patients.
Population characteristics	In Baylor Scott and White Discovery cohort, Black American patients with the median age of 58.2 ±10.89 White American patients with a median age of 60.06 ± 13.00, diagnosed with TNBC prior to sample collection were used in this study. Patients under the Roswell Park Validation cohort had the median age of 53.66 ± 14.54 in the Black Americans and 56.83 ± 15.19 in the white Americans.
Recruitment	De-identified breast cancer patient samples with at least 10-year follow-up and self-reported race, were obtained from Baylor Scott and White hospital (BSWH), Temple, Texas and Roswell Park Comprehensive Cancer Center, Buffalo, New York. The patients underwent Standard of Care treatment in these respective hospitals and the samples were collected with patient consent and respective IRB approvals and banked. The banked samples were de-identified and provided along with the metadata.
Ethics oversight	All the human studies were performed under IRB (Institutional Review Board) protocols 020-393 and 130559 (Baylor Scott and White Hospital), H-28445 (Baylor College of Medicine), H21060 (Georgia State University), 300009407 (University of Alabama at Birmingham) and Roswell Park Comprehensive Cancer Center. The approved BSW IRB protocol 020-393 waived the requirement of authorization based on 45 CFR 164.521(i)(2)(ii) and determined informed consent is not required as allowed under 45 CFR 46.116 (g). Patients from Roswell Park Comprehensive Cancer Center were consented. For patients from the Georgia cohort, the IRB H21060 from Georgia State University approved that patient consent will not be required since all samples used are archival and were de-identified to maintain patient privacy and anonymity.

Note that full information on the approval of the study protocol must also be provided in the manuscript.

Field-specific reporting

Please select the one below that is the best fit for your research. If you are not sure, read the appropriate sections before making your selection.

[x] Life sciences [ ] Behavioural & social sciences [ ] Ecological, evolutionary & environmental sciences

For a reference copy of the document with all sections, see nature.com/documents/nr-reporting-summary-flat.pdf

Life sciences study design

All studies must disclose on these points even when the disclosure is negative.

Sample size	Tissue cores from 57 tumors from Baylor Scott and white discovery dataset, 10 tumors from Baylor Scott and white validation dataset and 46 tumors from Roswell park dataset were used for this study. No prior power analysis was performed. Samples were chosen based on clinical features listed in table 1 and 2 in the manuscript
Data exclusions	We applied strict quality controls, excluding cores with low cell counts, limited tumor content, or partial cores from each patient cohort.
Replication	No replication of samples was performed. However, the main findings were repeated in independent clinical cohorts using othogonal methodologies.
Randomization	As this study focuses on racial disparity, randomization is not applicable. However, within each race groups, clinically balanced cohorts were analyzed without apriori bias.
Blinding	N/A

Reporting for specific materials, systems and methods

We require information from authors about some types of materials, experimental systems and methods used in many studies. Here, indicate whether each material, system or method listed is relevant to your study. If you are not sure if a list item applies to your research, read the appropriate section before selecting a response.

Materials & experimental systems

n/a	Involved in the study
	☒ Antibodies
☒	☐ Eukaryotic cell lines
☒	☐ Palaeontology and archaeology
☒	☐ Animals and other organisms
	☒ Clinical data
☒	☐ Dual use research of concern
☒	☐ Plants

Methods

n/a	Involved in the study
☒	☐ ChIP-seq
☒	☐ Flow cytometry
☒	☐ MRI-based neuroimaging

Antibodies

Antibodies used

1. Antibodies for Multiplex Immunofluorescence Analysis:
Primary antibodies (goat anti-CD31, R&D, AF3628; rabbit anti-CD163, Abcam, ab182422)
Secondary antibodies (Alexa Fluor 488-conjugated Donkey anti-Rabbit-IgG, Jackson ImmunoResearch, 711-545-152; Alexa Fluor 555-conjugated Donkey anti-Goat IgG, Thermo Scientific, A-21432)

2. List of antibodies and the metal tags used for Imaging Mass Cytometry (IMC) analysis:

Metal Tag	Antibody
143Nd All samples)	Vimentin
144Nd (All samples)	PLK1
145Nd (All samples)	AR
146Nd (All samples)	CD16
147Sm (All samples)	CD163
148Nd (All samples)	Pan Cytokeratin
149Sm (BSW2 validation and Roswell Park validation samples); 151Eu (BSW-Discovery samples)	CD31
150Nd (All samples)	PD-L1
151Eu (BSW2 validation and Roswell Park validation samples); 165Ho (BSW-Discovery samples)	PD-1
152Sm (All samples)	CD45
154Sm (All samples)	CD11c
155Gd (All samples)	FOXP3
156Gd (All samples)	CD4
158Gd (All samples)	E-Cadherin
159Tb (All samples)	CD68
160Gd (Roswell Park validation samples)	CD14
161Dy (All samples)	CD152/CTLA4
162Dy (BSW-Discovery and BSW2 validation samples); Pr141 (Roswell Park validation samples)	CD8a
162Dy (Roswell Park validation samples)	NOS2
163Dy (All samples)	VEGF
164Dy (BSW2 validation and Roswell Park validation samples)	MPO
165Ho (BSW2 validation and Roswell Park validation samples); 164Dy (BSW-Discovery samples)	HIF1a
166Er (All samples)	CD45RA
167Er (All samples)	Granzyme B
168Er (All samples)	Ki-67
169Tm (Roswell Park validation samples)	Arginase-1
170Er (All samples)	CD3
173Yb (All samples)	CD45RO
175Lu (All samples)	KIFC1
176Yb (All samples)	pHH3
Nd142 (Roswell Park validation samples)	CD20

All the antibodies were prepared according to the manufacturer's protocols provided by Standard BioTools, measured for absorbance, and stored in Candor PBS Antibody Stabilization solution (Candor Bioscience) at 4°C.

Refer supplementary tables 2,3 and 4.

Validation

Species specific validation was ensured through statements from each manufacturer's website.

Clinical data

Policy information about clinical studies

All manuscripts should comply with the ICMJE guidelines for publication of clinical research and a completed CONSORT checklist must be included with all submissions.

Clinical trial registration	*Provide the trial registration number from ClinicalTrials.gov or an equivalent agency.*
Study protocol	*Note where the full trial protocol can be accessed OR if not available, explain why.*
Data collection	*Describe the settings and locales of data collection, noting the time periods of recruitment and data collection.*
Outcomes	*Describe how you pre-defined primary and secondary outcome measures and how you assessed these measures.*

Plants

Seed stocks	*Report on the source of all seed stocks or other plant material used. If applicable, state the seed stock centre and catalogue number. If plant specimens were collected from the field, describe the collection location, date and sampling procedures.*
Novel plant genotypes	*Describe the methods by which all novel plant genotypes were produced. This includes those generated by transgenic approaches, gene editing, chemical/radiation-based mutagenesis and hybridization. For transgenic lines, describe the transformation method, the number of independent lines analyzed and the generation upon which experiments were performed. For gene-edited lines, describe the editor used, the endogenous sequence targeted for editing, the targeting guide RNA sequence (if applicable) and how the editor was applied.*
Authentication	*Describe any authentication procedures for each seed stock used or novel genotype generated. Describe any experiments used to assess the effect of a mutation and, where applicable, how potential secondary effects (e.g. second site T-DNA insertions, mosiacism, off-target gene editing) were examined.*
