# Supplementary material for: Integrative spatial omics reveals distinct tumor-promoting multicellular niches and immunosuppressive mechanisms in Black American and White American patients with TNBC
Source: Nat Commun. 2025 Jul 17;16:6584. doi: 10.1038/s41467-025-61034-3 (PMC12271405; doi:10.1038/s41467-025-61034-3)
Supplement: Supplementary file 4 — Source Data 1 [file 41467_2025_61034_MOESM4_ESM.zip › SFig2.pdf]

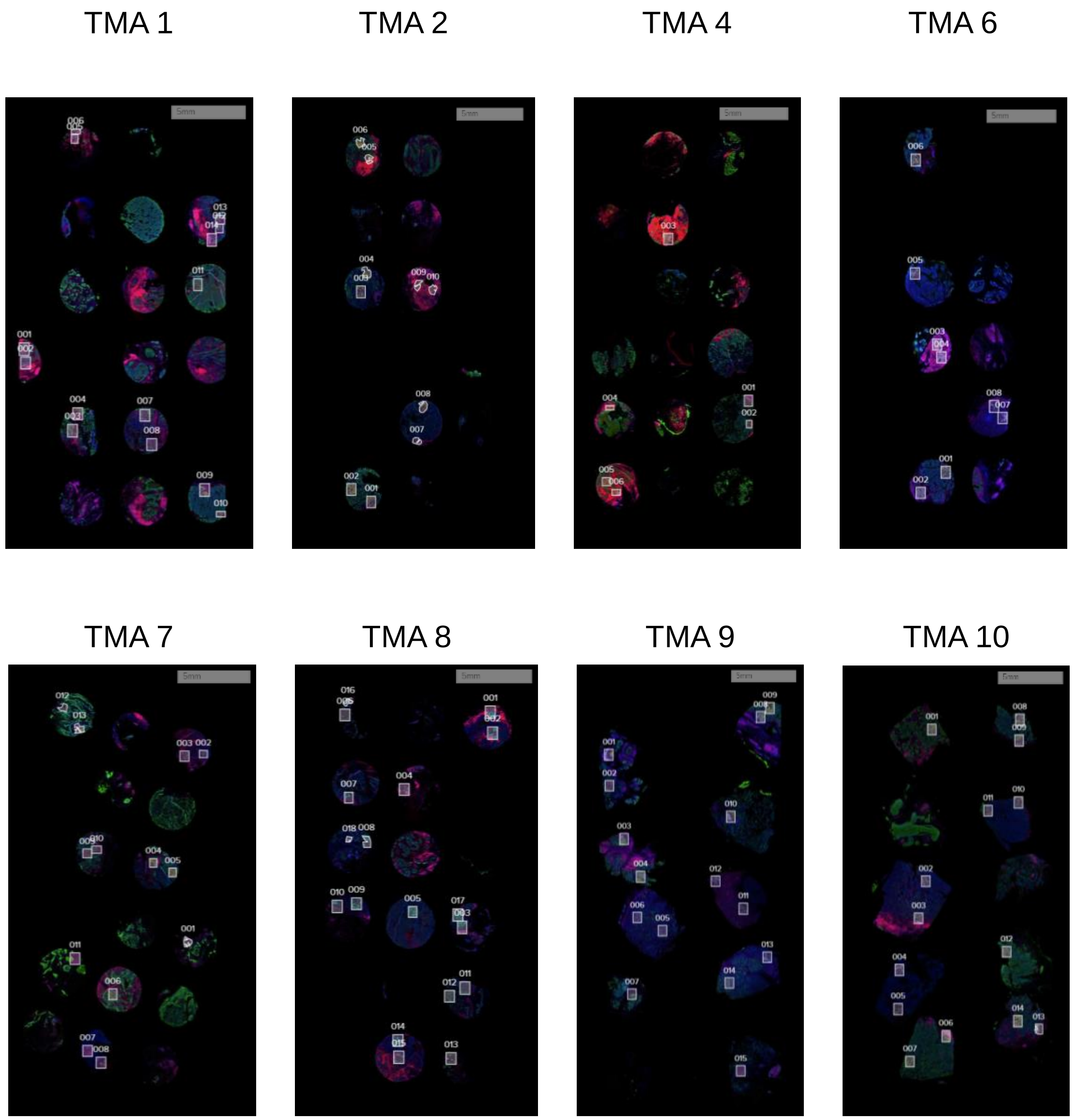

Photomicrographs of tissue microarrays

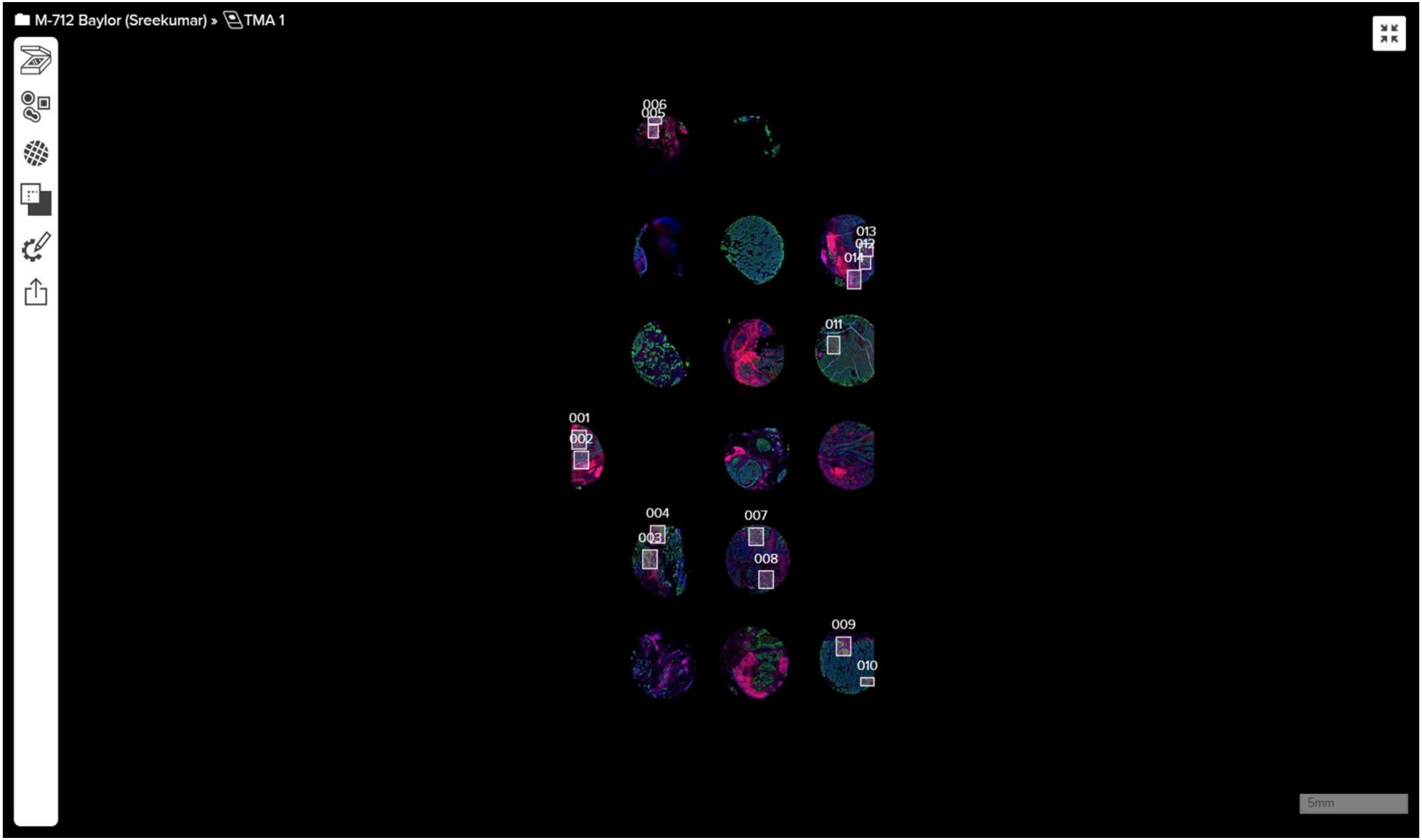
M-712 Baylor (Sreekumar) » TMA 1
006
005
013
012
014
011
001
002
004
003
007
008
009
010
5mm

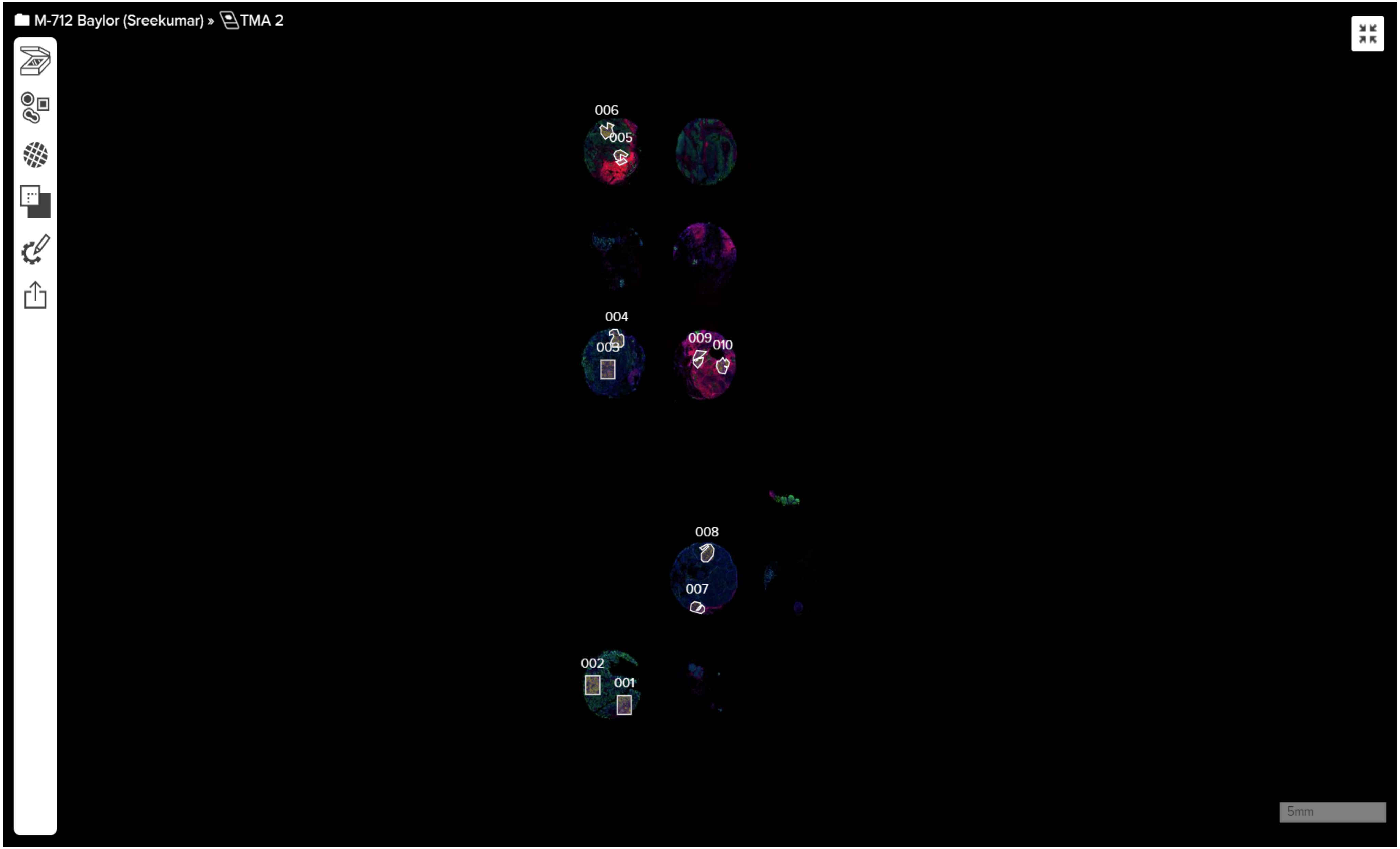
M-712 Baylor (Sreekumar) » TMA 2
006
005
004
003
009
010
008
007
002
001
5mm

M-712 Baylor (Sreekumar) » TMA 4

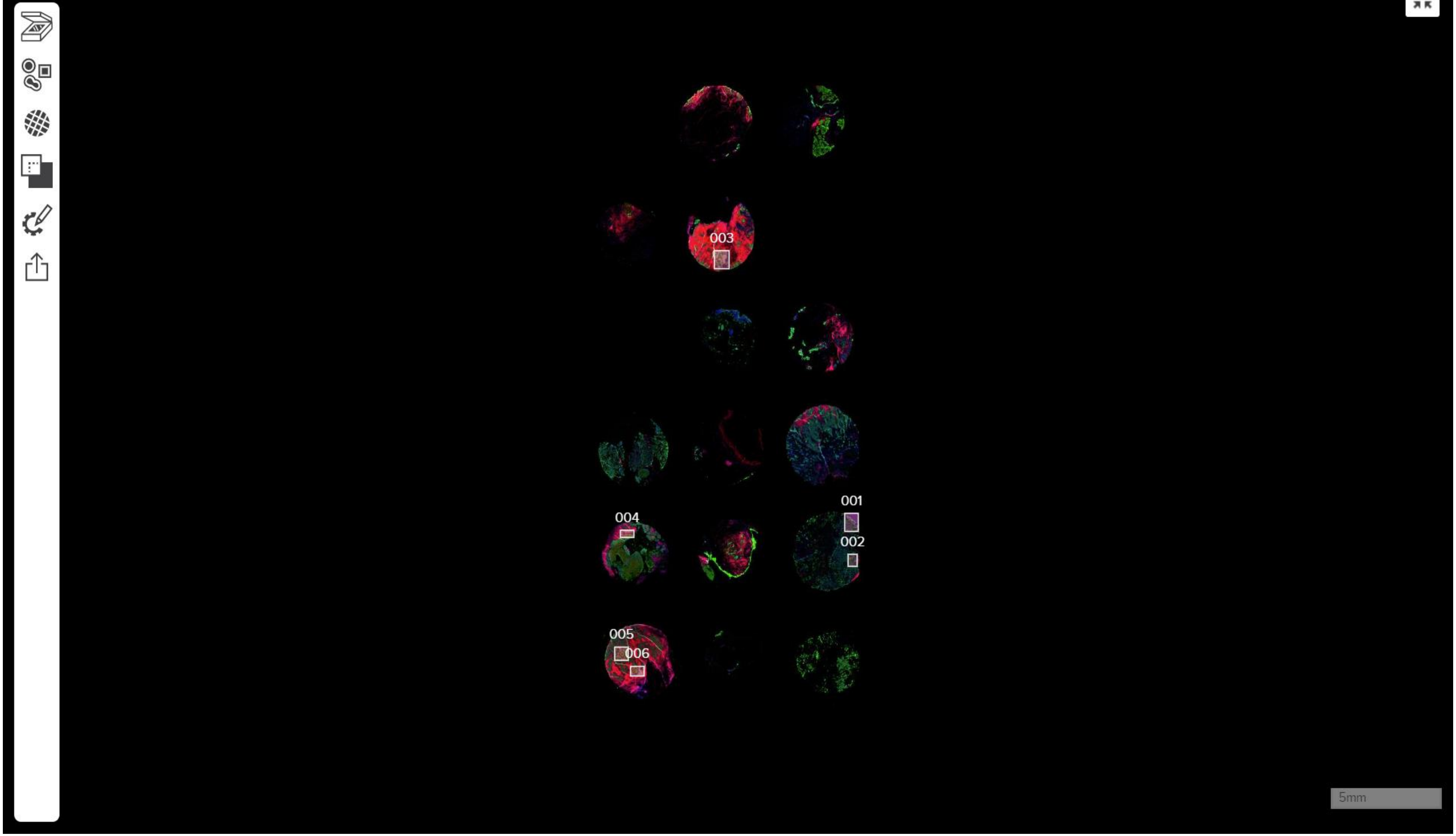

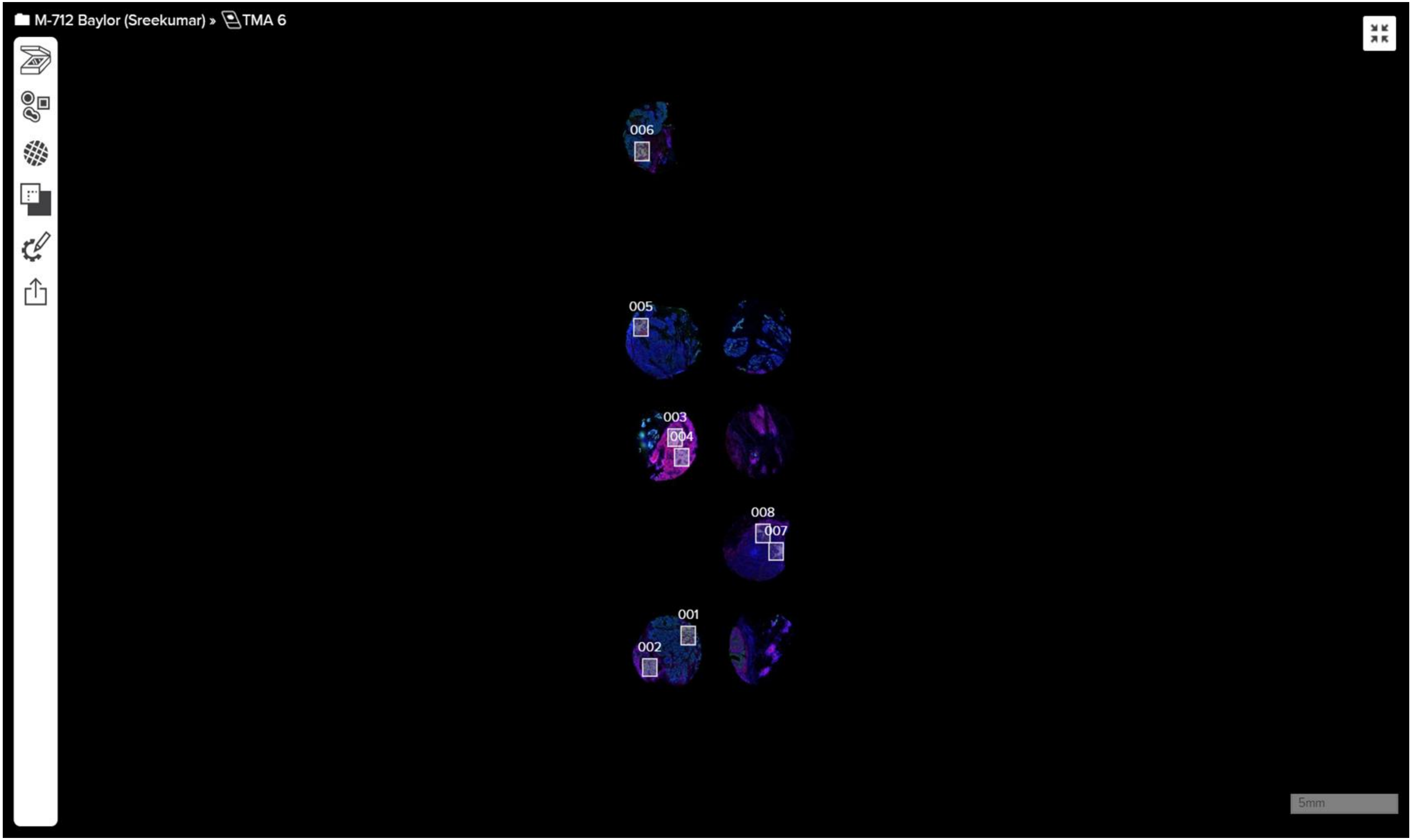
M-712 Baylor (Sreekumar) » TMA 6
006
005
003
004
008
007
001
002
5mm

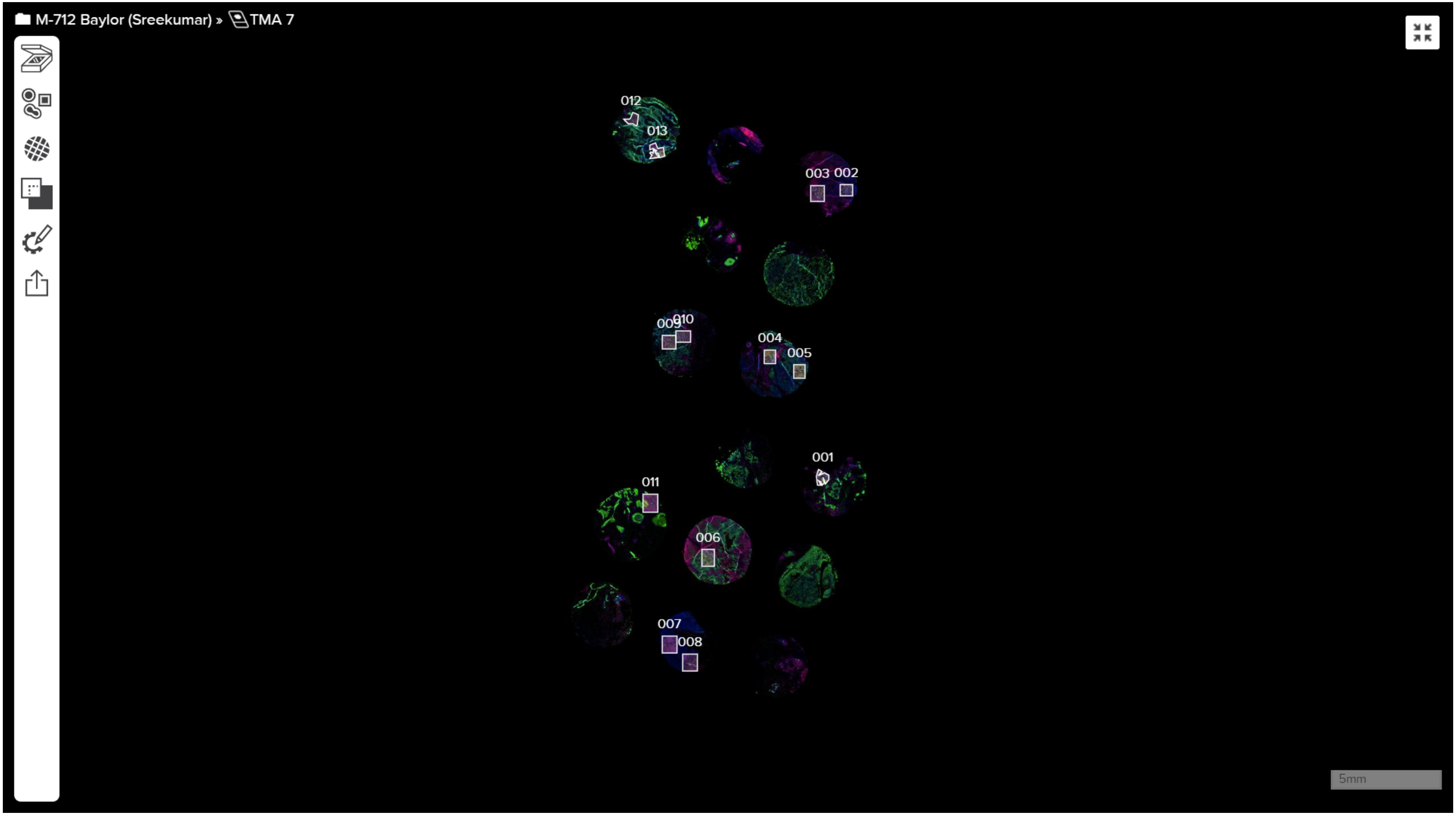
M-712 Baylor (Sreekumar) » TMA 7
012
013
003 002
009
010
004
005
001
011
006
007
008
5mm

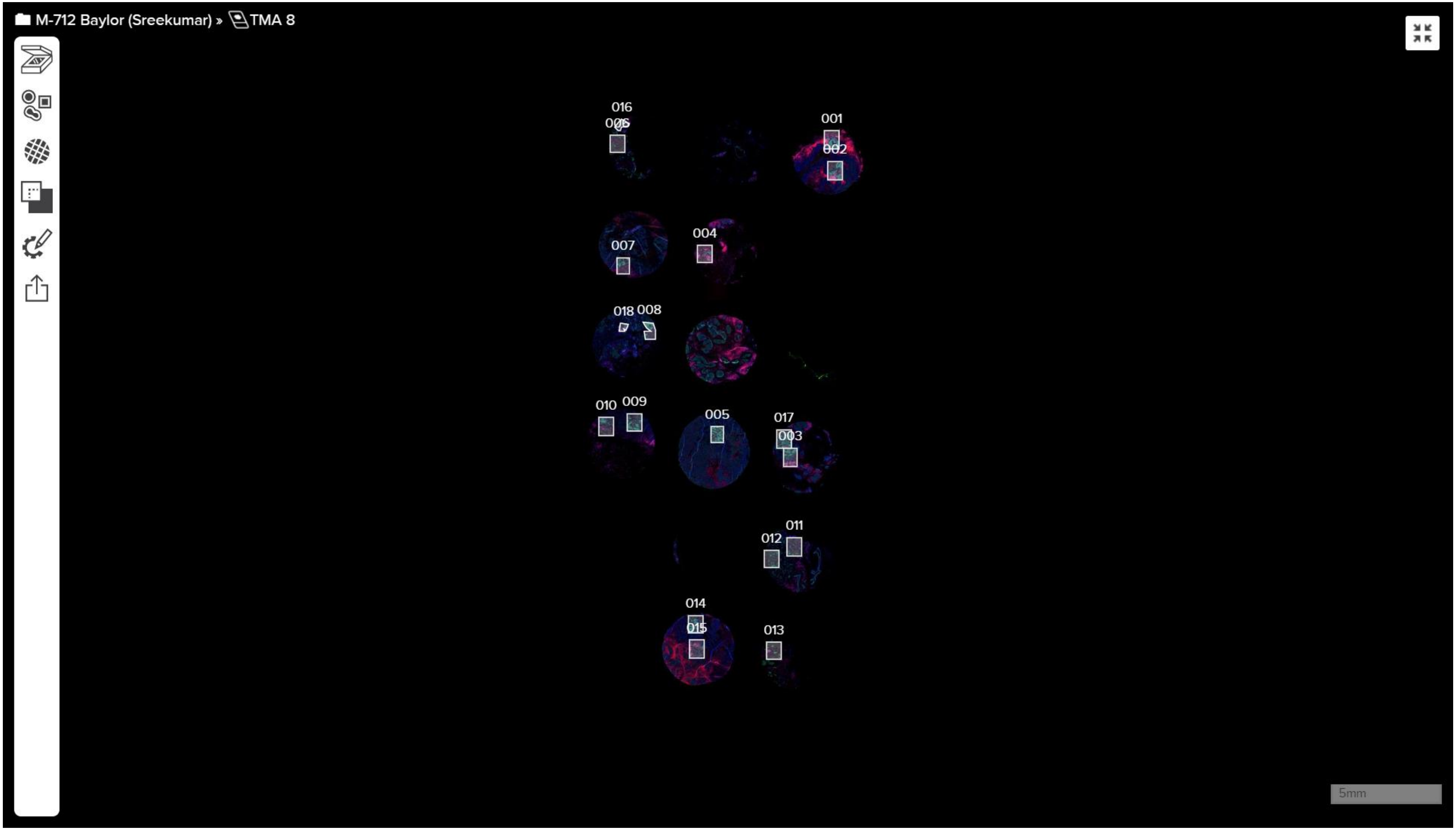
M-712 Baylor (Sreekumar) » TMA 8
016
006
001
002
007
004
018
008
010
009
005
017
003
011
012
014
015
013
5mm

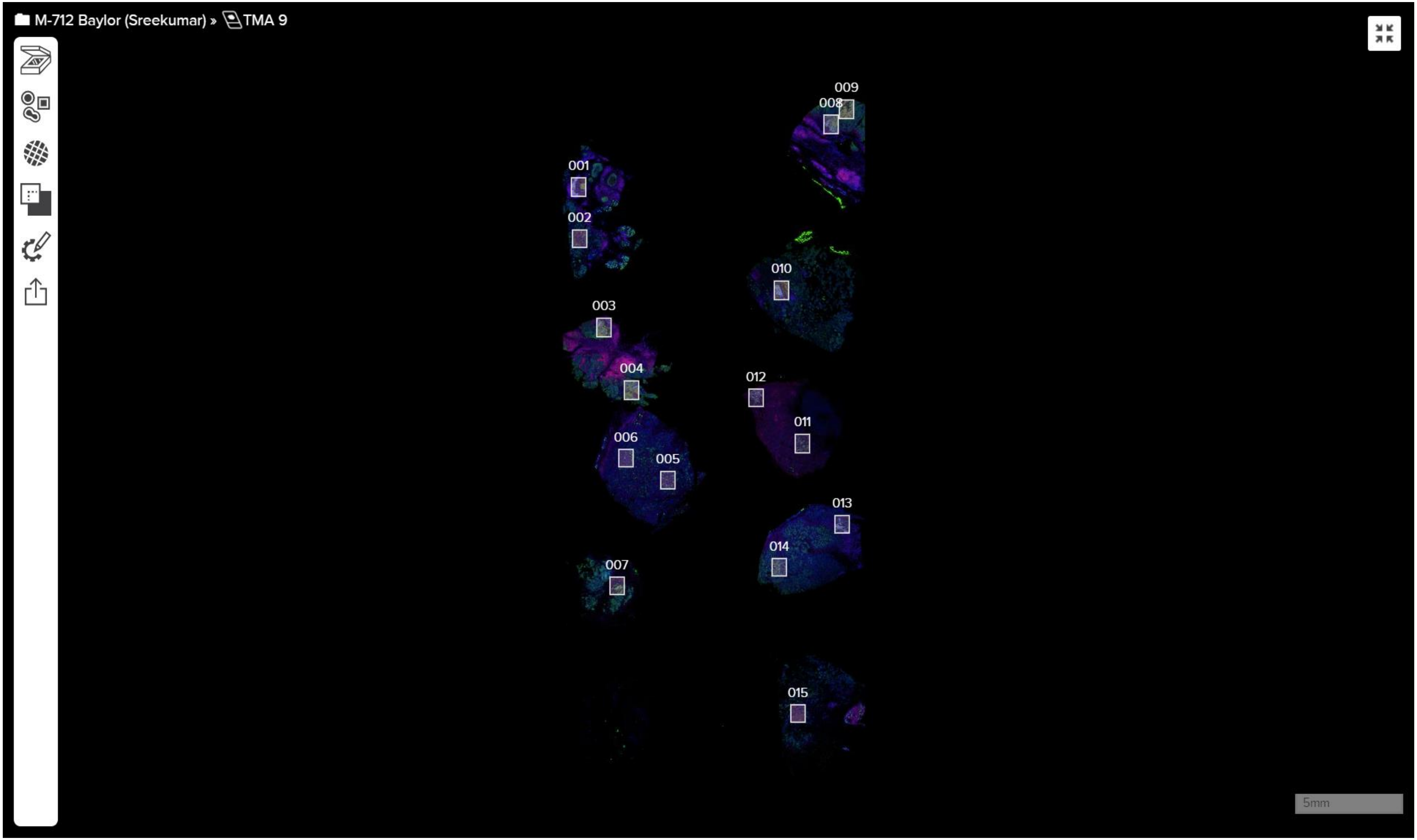
M-712 Baylor (Sreekumar) » TMA 9
009
008
001
002
010
003
004
012
011
006
005
013
014
007
015
5mm

M-712 Baylor (Sreekumar) » TMA 10

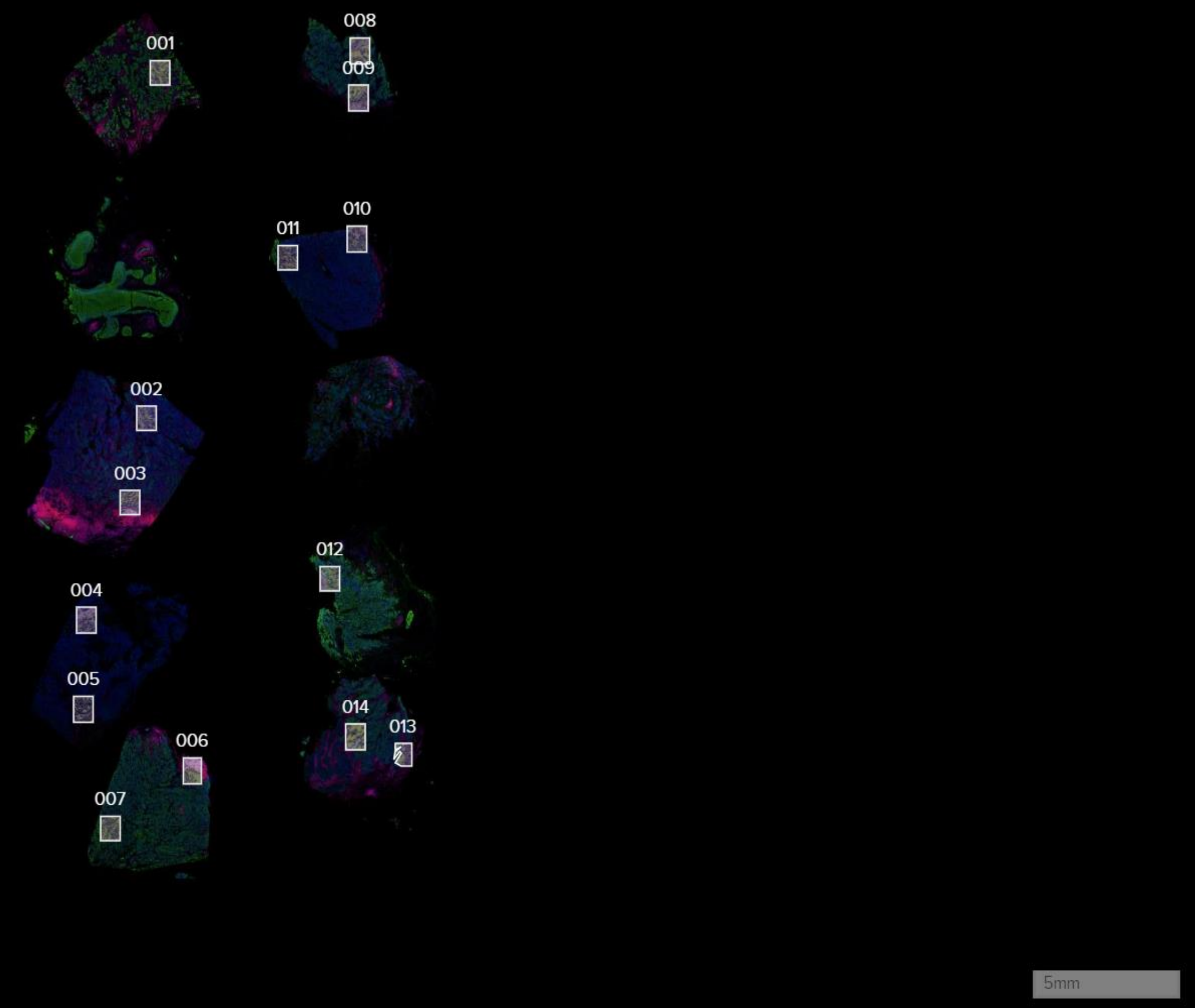

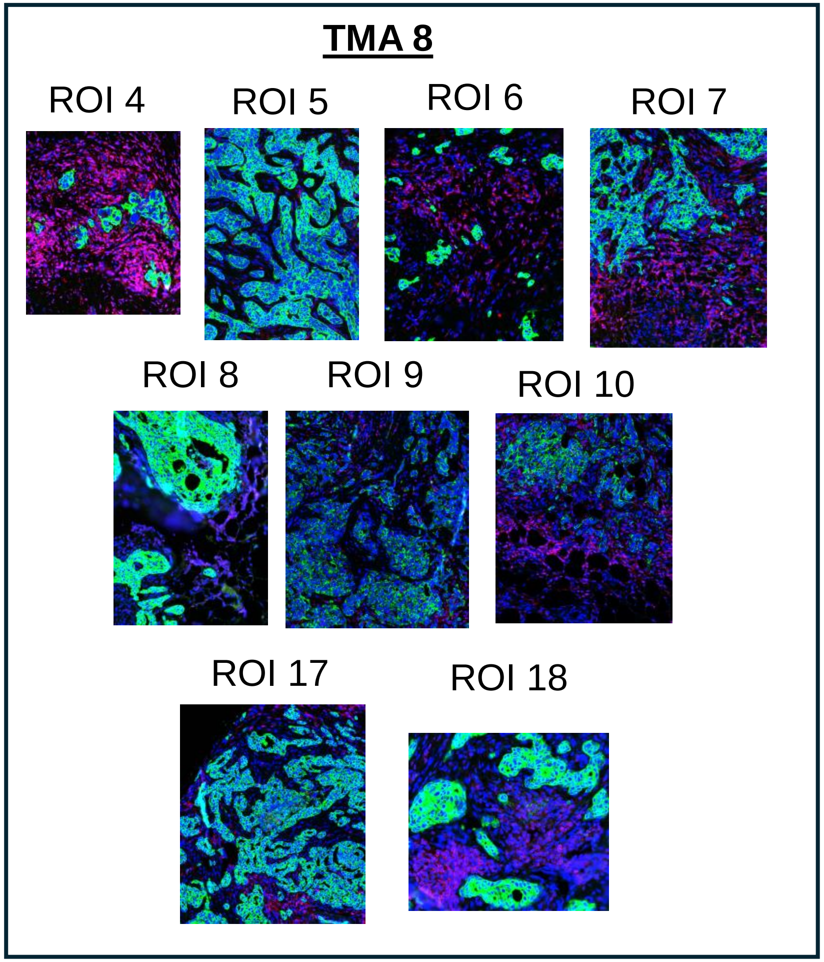
TMA 8
ROI 4
ROI 5
ROI 6
ROI 7
ROI 8
ROI 9
ROI 10
ROI 17
ROI 18

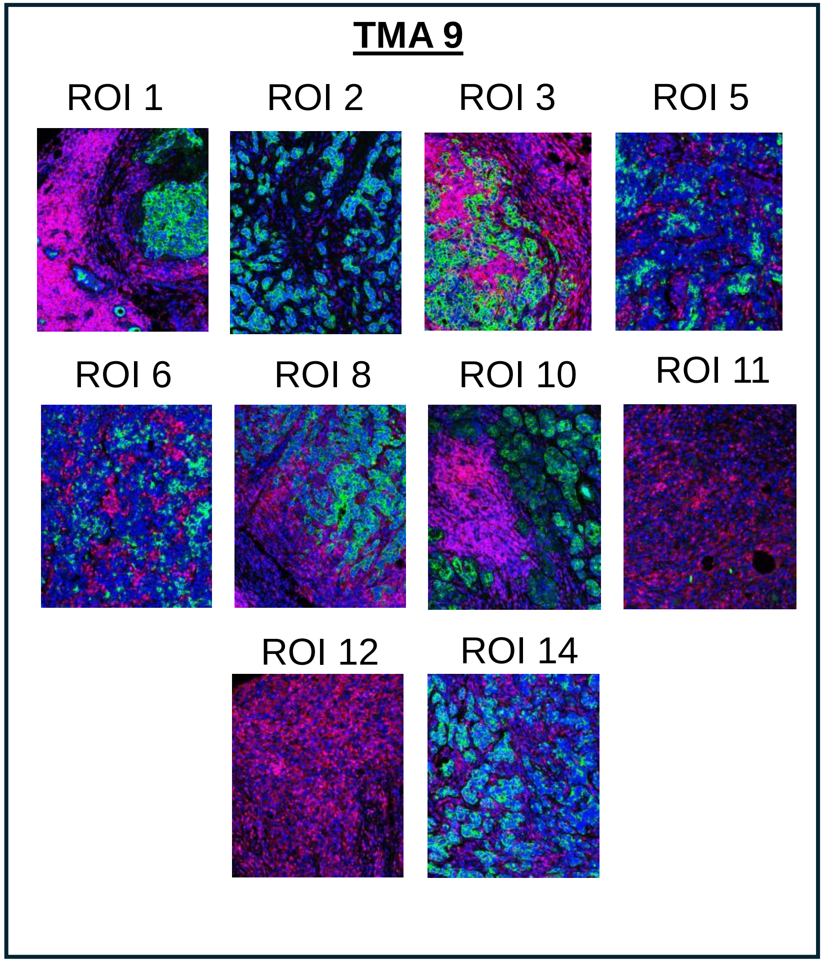
TMA 9
ROI 1
ROI 2
ROI 3
ROI 5
ROI 6
ROI 8
ROI 10
ROI 11
ROI 12
ROI 14

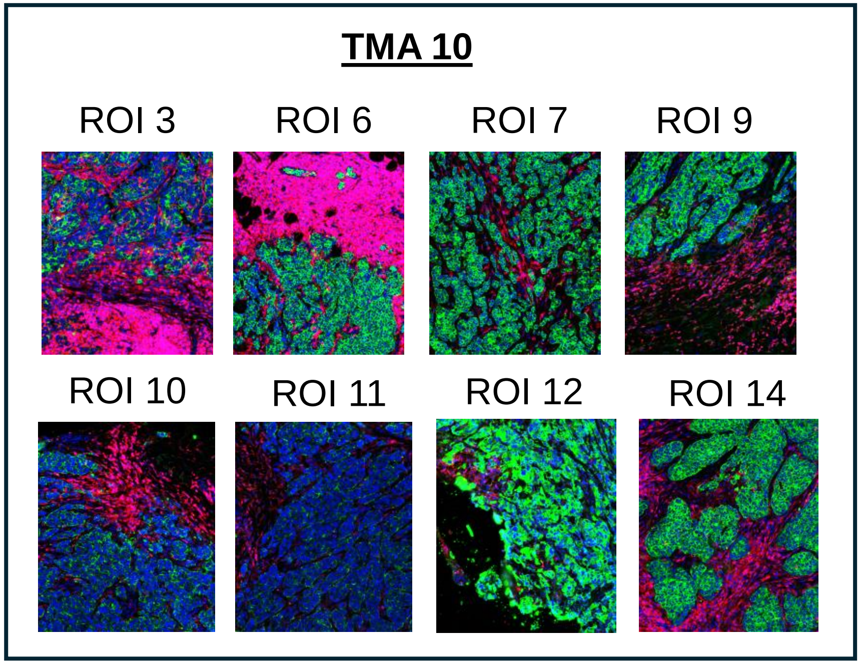
TMA 10
ROI 3
ROI 6
ROI 7
ROI 9
ROI 10
ROI 11
ROI 12
ROI 14

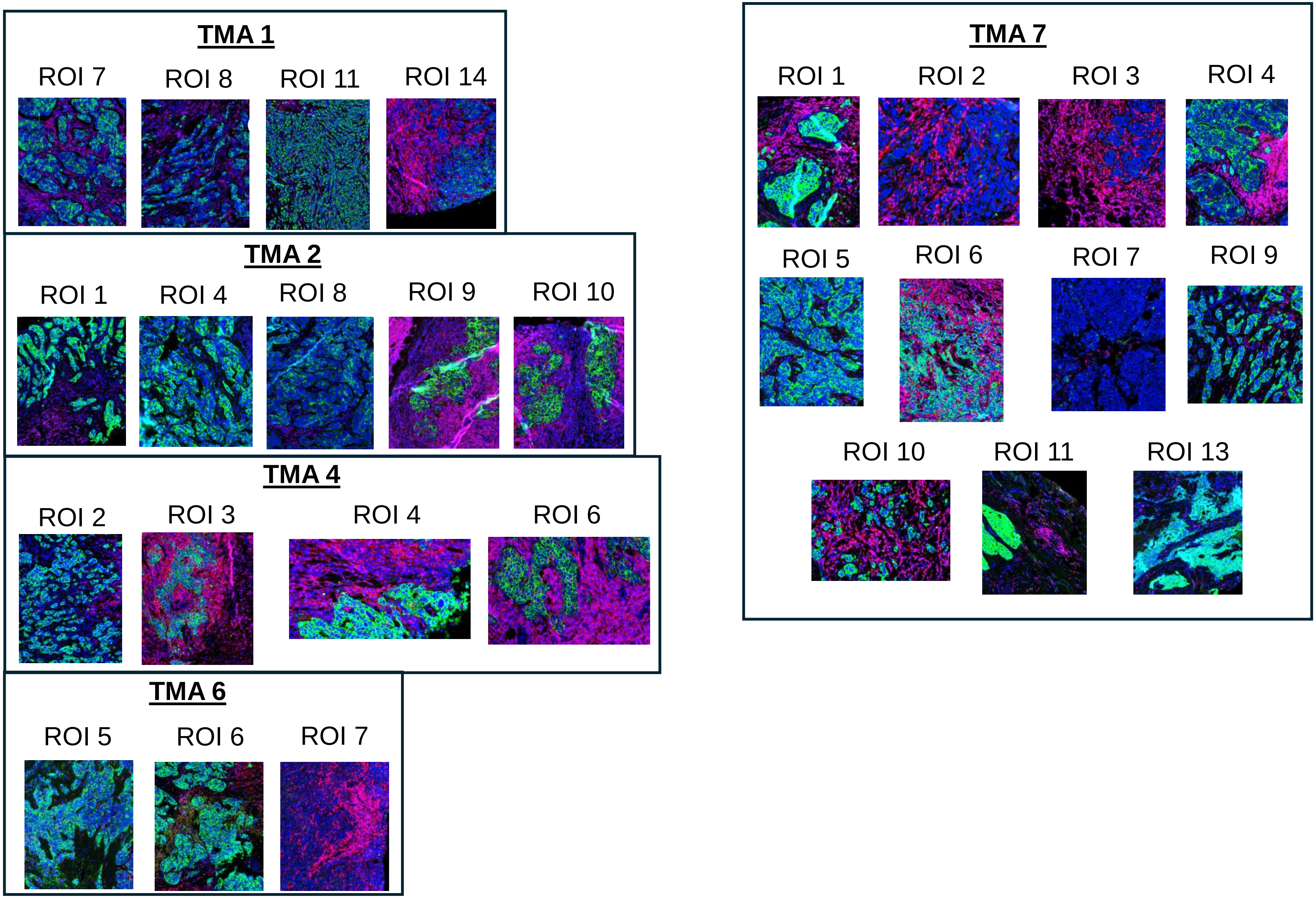
TMA 1
ROI 7
ROI 8
ROI 11
ROI 14
TMA 2
ROI 1
ROI 4
ROI 8
ROI 9
ROI 10
TMA 4
ROI 2
ROI 3
ROI 4
ROI 6
TMA 6
ROI 5
ROI 6
ROI 7
TMA 7
ROI 1
ROI 2
ROI 3
ROI 4
ROI 5
ROI 6
ROI 7
ROI 9
ROI 10
ROI 11
ROI 13
